# Retrotransposon addiction promotes centromere function via epigenetically activated small RNAs

**DOI:** 10.1038/s41477-024-01773-1

**Published:** 2024-09-02

**Authors:** Atsushi Shimada, Jonathan Cahn, Evan Ernst, Jason Lynn, Daniel Grimanelli, Ian Henderson, Tetsuji Kakutani, Robert A. Martienssen

**Affiliations:** 1 Howard Hughes Medical Institute, Cold Spring Harbor Laboratory, New York, NY USA; 2 DIADE, IRD-CIRAD, Université de Montpellier, Montpellier, France; 3 Department of Plant Sciences, Cambridge University, Cambridge, UK; 4 Faculty of Science, The University of Tokyo, Tokyo, Japan

**Keywords:** RNAi, Epigenetics

## Abstract

Retrotransposons have invaded eukaryotic centromeres in cycles of repeat expansion and purging, but the function of centromeric retrotransposons has remained unclear. In *Arabidopsis*, centromeric *ATHILA* retrotransposons give rise to epigenetically activated short interfering RNAs in mutants in *DECREASE IN DNA METHYLATION1* (*DDM1*). Here we show that mutants that lose both DDM1 and RNA-dependent RNA polymerase have pleiotropic developmental defects and mis-segregate chromosome 5 during mitosis. Fertility and segregation defects are epigenetically inherited with centromere 5, and can be rescued by directing artificial small RNAs to *ATHILA5* retrotransposons that interrupt tandem satellite repeats. Epigenetically activated short interfering RNAs promote pericentromeric condensation, chromosome cohesion and chromosome segregation in mitosis. We propose that insertion of *ATHILA* silences centromeric transcription, while simultaneously making centromere function dependent on retrotransposon small RNAs in the absence of DDM1. Parallels are made with the fission yeast *Schizosaccharomyces* *pombe*, where chromosome cohesion depends on RNA interference, and with humans, where chromosome segregation depends on both RNA interference and HELLS^DDM1^.

## Abstract (web summary)

Centromeric satellite repeats on *Arabidopsis* chromosome 5 are interrupted by *ATHILA5* retrotransposons, and cohesion is compromised in *ddm1* chromatin remodelling mutants that have also lost RNAi. Mis-segregation is epigenetically inherited but can be rescued by *ATHILA5* small RNA.

## Main

Eukaryotic centromeres are usually composed of repetitive sequences with a unique chromatin composition that includes the centromeric histone H3 variant CENH3 (ref. ^1^). CENH3 assembles the kinetochore, a large protein complex that attaches the chromosome to the spindle^1^. The positioning of CENH3 is thought to be epigenetically defined by surrounding pericentromeric heterochromatin—chromosomal material that remains condensed in interphase^2,3^. Pericentromeric heterochromatin is also responsible for sister chromatid cohesion at mitosis, which ensures segregation of sister chromatids to each daughter cell during anaphase^1^. In many eukaryotes, these repetitive centromere sequences are composed of rapidly evolving tandem satellite repeats^1,2^. In plants, animals and fungi, satellite repeats are interspersed with specific classes of retrotransposons but the function, if any, of these retrotransposons has remained obscure^1^.

DNA methylation and RNA interference (RNAi) are important epigenetic pathways for both transcriptional and post-transcriptional gene silencing. In the model plant *Arabidopsis* *thaliana*, DNA methylation is required to silence transposons, and can be triggered by RNAi through a pathway called RNA-dependent DNA methylation (RdDM). RdDM relies on 24-nt siRNAs produced by RNA POLYMERASE IV, RNA-DEPENDENT RNA POLYMERASE 2 (RDR2) and DICER-LIKE 3 (DCL3)^4,5^. These 24-nt small RNAs bind to ARGONAUTE 4 (AGO4) and related proteins, which are thought to recruit DNA methyltransferases to RNA POLYMERASE V, along with other chromatin-modifying enzymes^4,5^. In organisms without DNA methylation, such as *Drosophila* *melanogaster*, *Caenorhabditis* *elegans* and the fission yeast *S.* *pombe*, RNAi guides histone modifications, notably dimethylation of histone H3 lysine-9 (refs. ^6–8^), which plays a major role in centromere cohesion. For this reason, *S.* *pombe* RNAi mutants have strong defects in chromosome segregation^9,10^. However, in *Arabidopsis*, such mitotic defects are very mild, or not apparent, when components of the canonical RdDM pathway are mutated, despite the complete loss of the 24-nt small RNA^11^.

DNA methylation can be maintained in the absence of RdDM by the DDM1 SWI2/SNF2 chromatin remodeler, but mutants retain fertility and normal chromosome segregation despite substantial demethylation of centromeric satellite repeats^12^. Although RdDM-mediated DNA methylation is required for transcriptional gene silencing, *Arabidopsis* possesses another RNAi pathway for post-transcriptional gene silencing, which generates 21-nt or 22-nt siRNAs via *RDR6*-*DCL2/DCL4-AGO1/AGO7* and silences euchromatic genes, transgenes and viral RNAs^13^. We previously identified a class of 21-nt epigenetically activated short interfering RNAs (easiRNAs) derived from transposable elements in *ddm1* mutants^14^, which have elevated transcription of transposons^15^. Similar small RNAs are found in *ddm1-*like double mutants in maize, although mutant embryos fail to germinate in this species^16^. We rationalized that small RNAs might compensate for the loss of DNA methylation in *ddm1* mutants, and set out to determine the developmental and chromosomal consequences of removing RNAi in the absence of DNA methylation.

## Epigenetic defects map to centromere 5 in RNAi and *ddm1* mutants

**Fig. 1:**
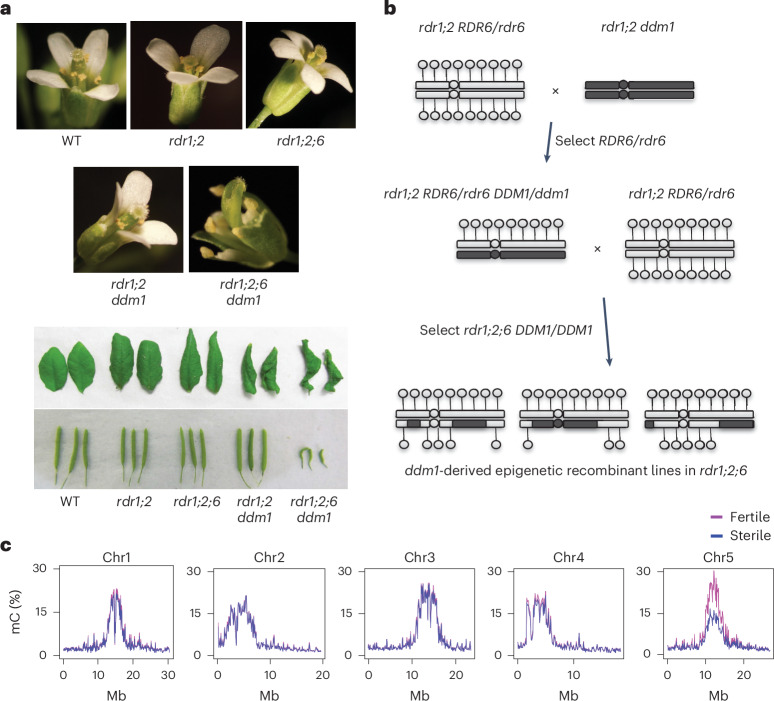
Fertility and floral defects of *rdr1;2;6 ddm1* map to hypomethylated centromere 5. **a**, Developmental defects of double, triple and quadruple mutants in RNA-dependent RNA polymerase (*rdr1*, *rdr2*, *rdr6*) and DNA methylation (*ddm1*) in floral organ identity, leaf shape and fertility (silique length). **b**, Crossing scheme for constructing *ddm1*-derived epigenetic recombinant lines in an *rdr1 rdr2 rdr6* background. Hypomethylated chromosomal regions derived from *ddm1* mutants are inherited epigenetically in *DDM1*/*DDM1* progeny, and are indicated in dark grey. Methylated cytosines are indicated as lollipops. **c**, Methylome analysis by WGBS of pooled fertile (pink, pool of 10 plants) and sterile (blue, pool of 10 plants) epigenetic recombinant lines (from **b**) indicates reduced cytosine methylation (mC) in the pericentromeric regions of chromosome 5. Chromosome co-ordinates are given in megabases (Mb).

**Extended Data Fig. 1:**
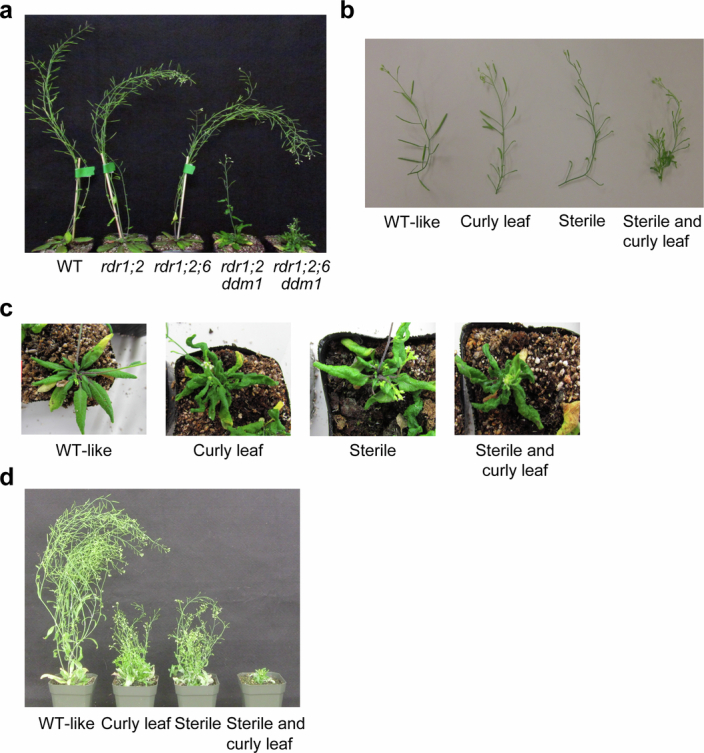
Phenotypes of *rdr1;2;6 ddm1* and of *ddm1* epiRILs in an *rdr1;2;6* background. **a**, Plant stature phenotypes in the indicated genotypes. **b**-**d**, The phenotypes of *ddm1* epiRILs were classified into 4 groups (WT-like, Curly leaf, Sterile, Sterile and Curly leaf). Panels show (**b**) siliques, (**c**) leaves, and (**d**) stature of each group.

The biosynthesis of 21- or 22-nt easiRNAs is dependent on RDR6 (*AT3G49500*)^17,18^, which is partially redundant with RDR1 (*AT1G14790*)^19^, whereas RDR2 (*AT4G11130*) contributes to RNA-dependent DNA methylation via 24-nt siRNAs^20,21^. We have previously shown that *ddm1* (*AT5G66750*) mutants additionally bearing mutations of all three RNA-dependent RNA polymerase genes (*rdr1 rdr2 rdr6 ddm1*, hereafter *rdr1;2;6 ddm1*), have severe developmental defects, unlike *ddm1*, *rdr1;2 ddm1* or *rdr1;2;6* alone^17,18^. *rdr1;2;6 ddm1* mutants exhibit pleiotropic developmental defects such as infertility, short stature, slow growth, curly leaves and flowers with additional stamens and missing organs (Fig. 1a and Extended Data Fig. 1a). By contrast, no conspicuous phenotype is observed in *rdr1;2 or rdr1;2;6* mutants, while in *rdr1;2; ddm1* mutants only vegetative phenotypes, such as curly leaves and short stature, are observed (Fig. 1a and Extended Data Fig. 1a). Thus, *RDR6* activity is essential for fertility and floral organ development in the absence of DNA methylation^18,22^. Importantly, backcrosses to *rdr1;2;6* triple mutants demonstrated that these phenotypes were inherited epigenetically when *DDM1* function was restored in the absence of RDRs (Fig. 1b).

**Extended Data Fig. 2:**
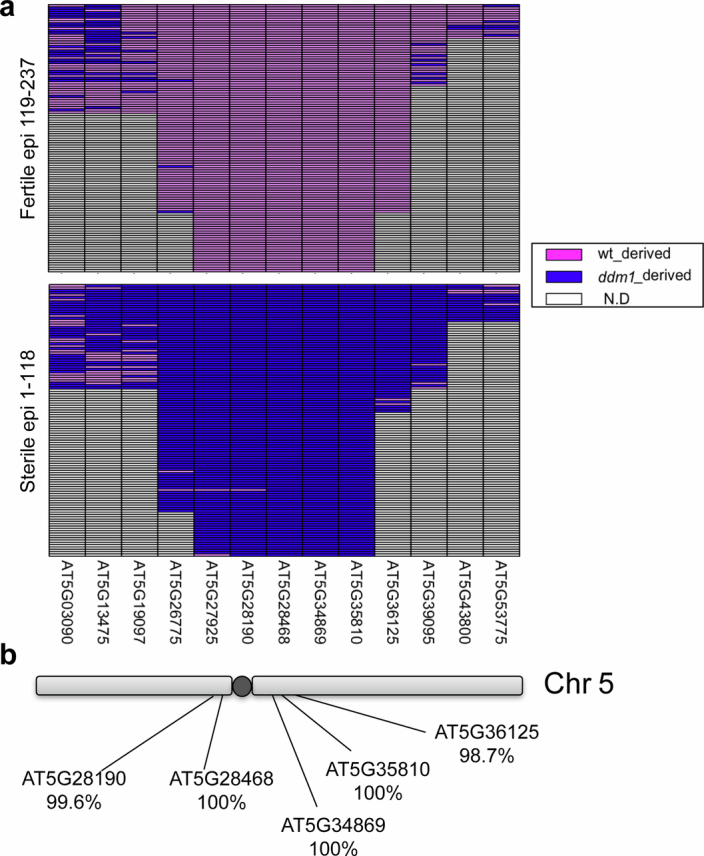
Epigenetic mapping of the sterility phenotype in *rdr1;2;6 ddm1.* **a**, DNA was extracted from fertile and sterile *ddm1* epigenetic recombinant lines (sterile;epi 1-118, fertile;epi 119-237) in *rdr1;2;6* background, and DNA methylation at the indicated transposable elements (AT5G03090-AT5G53775) was assessed by McrBC-based PCR analysis. Upper and lower panels indicate methylation maps of fertile and sterile epigenetic recombinant lines, respectively. Chromosomal regions derived from WT and *ddm1* are coloured in pink and blue, respectively. **b**, An epigenetic linkage map of the sterility phenotype in *rdr1;2;6*. Linkage between DNA hypomethylation with the sterile phenotype is indicated below TE gene names.

Because mutants defective in DNA methylation have been shown to suffer from developmental phenotypes due to mis-expression of individual genes^23–25^, we hypothesized that there might be a causative locus that is silenced by *RDR6*-dependent 21-nt siRNAs in *ddm1* mutants. To identify this locus, we performed genetic mapping by generating *ddm1*-derived epigenetic recombinant lines in an *rdr1;2;6* mutant background. Because the loss of DNA methylation in *ddm1* is epigenetically inherited, especially in an *rdr2* mutant background^21^, *ddm1*-derived chromosomes remain hypomethylated even after backcrossing to wild type (WT). This allowed us to identify which chromosomal region or regions were responsible for the phenotype. Epigenetic recombinant lines in an *rdr1;2;6* background were generated in the crossing scheme shown in Fig. 1b. Plants were classified into four groups depending on their phenotypes: (1) WT-like, (2) curly leaf, (3) sterile and (4) both sterile and curly leaf (Extended Data Fig. 1b–d). The sterile phenotype was always associated with the floral defect (Extended Data Fig. 1b–d), suggesting that these defects may arise from the same dominant mutation. We performed whole-genome bisulfite sequencing (WGBS) analysis to compare genome-wide DNA methylation levels between sterile and fertile plants from these backcrosses. This analysis demonstrated that the sterile and floral phenotypes were linked to the hypomethylated centromeric region of chromosome 5, derived from *ddm1* (Fig. 1c). We performed fine mapping using McrBC, a restriction enzyme that digests only methylated DNA, and amplification by PCR, to determine whether a given chromosomal region was *ddm1*derived or WT derived^26^. In this way, the causative locus was mapped to the interval between *AT5G28190* and *AT5G36125* (Extended Data Fig. 2). Although we examined more than 200 individuals, we could not narrow down this causative interval further because of the low frequency of meiotic crossovers in centromeric regions^27^.

## An *ATHILA5* retrotransposon promotes centromere function

**Fig. 2:**
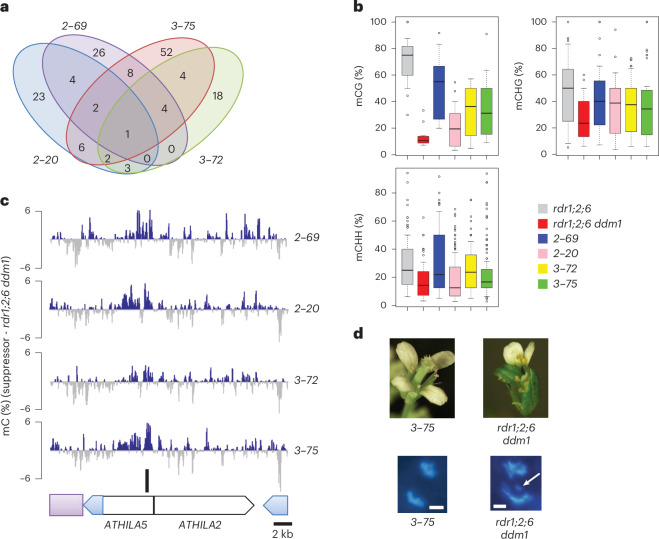
Epiallelic suppressors gain DNA methylation at an *ATHILA5* retrotransposon in centromere 5. **a**, Venn diagram of shared, hypermethylated DMRs in four independent *rdr1;2;6 ddm1* suppressors (*2–69*, *2–20*, *3–72*, *3–75*) on chromosome 5. **b**, Boxplot analyses of DNA methylation levels at each covered cytosine in the uniquely shared 1 kb hypermethylated DMR in each genotype. Data are presented as median (black line), lower and upper quartiles (box) ± 0.5 × interquartile range (whiskers) and outliers (open circles). **c**, Uniquely shared DMR (black bar) corresponds to *Cen5-ATHILA5* (blue long terminal repeats), which is embedded in cen180 satellite repeats (purple box) and interrupted by *ATHILA2*. Genome browser tracks display DNA methylation gains (blue) and losses (grey) in the 26 kb region in each suppressor line relative to *rdr1;2;6 ddm1*. **d**, Floral and chromosomal phenotypes of *rdr1;2;6 ddm1* mutants are rescued by epiallelic suppressor *3–75*. Mitotic chromosomes in root tip anaphase cells were stained with DAPI. A mis-segregating chromosome is indicated by a white arrow (scale bar, 2 µm, estimated from magnification).

**Extended Data Fig. 3:**
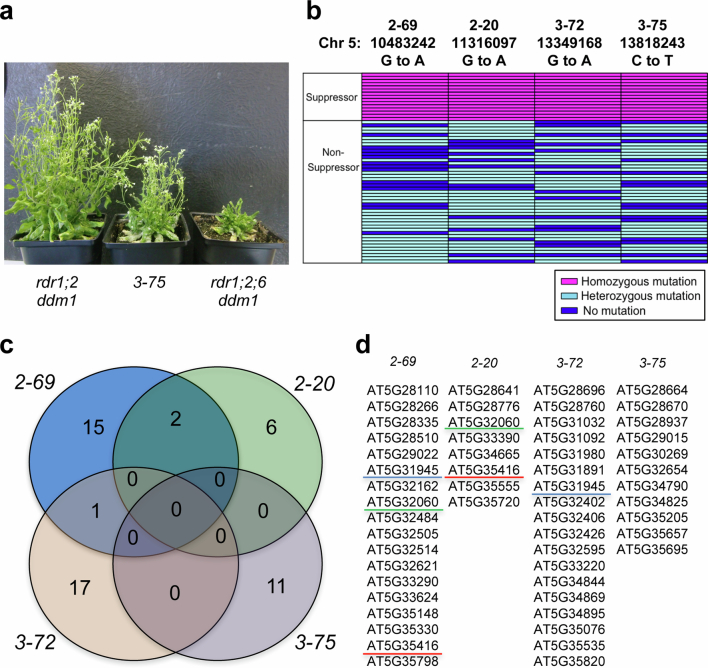
Genetic mapping of linked mutations in EMS suppressor lines. **a**, EMS suppressor 3-75 rescued the phenotype of *rdr1;2;6 ddm1*. **b**, CAPS analysis using EMS-induced SNPs was performed in M2 progeny segregating suppressors and non-suppressors. We focused on chromosome 5 centromeric region where the sterility defect mapped (Extended Data Fig. 2). SNPs used in the analysis are shown above the panel. Cells in the table are coloured by pink, light blue and blue, to indicate individuals bearing homozygous SNP, heterozygous SNP and no SNP, respectively. Each suppressor was recessive and tightly linked to Cen5. **c**, Venn diagram of mutations detected on chromosome 5 centromeric regions in EMS suppressors. **d**, A list of mutations introduced into chromosome 5 centromeric regions in *rdr1;2;6 ddm1* suppressors. Genes underlined with the same colour represent mutated genes in more than one suppressor.

Taking an alternative approach, we performed mutagenesis with ethyl methanesulfonate (EMS) to obtain suppressors that rescue the fertility defect in *rdr1;2;6 ddm1* mutants. Four genetic suppressors were isolated, which also rescued the short stature and floral developmental defects (Extended Data Fig. 3a). Whole-genome sequencing of pooled sterile and fertile segregants revealed that the suppressors were linked to single nucleotide polymorphisms (SNPs) on centromere 5 (Extended Data Fig. 3b), but curiously, there were no commonly mutated genes among the four suppressors and most of the introduced SNPs were in transposable elements (Extended Data Fig. 3c,d). Along with nucleotide substitutions, EMS mutagenesis is also capable of inducing changes in cytosine methylation, resulting in epialleles^28–30^. EMS-induced epialleles of *SUPERMAN*, for example, gain DNA methylation in the promoter region and behave like *superman* mutants without any change in the DNA sequence^28,30^. This led us to consider the possibility that suppression might be caused by epigenetic modification rather than nucleotide substitution. Therefore, we performed WGBS of pooled fertile and sterile segregants and DMR (differentially methylated regions) analysis using the TAIR10 assembly of the *Arabidopsis* Col-0 genome. This revealed a single hypermethylated locus in centromere 5 common to all four suppressors (Fig. 2a,b). This locus corresponds to the 5′ region of the *ATHILA5* retrotransposon *Cen5-ATHILA5* (Fig. 2c).

The vegetative and infertility phenotypes of *rdr1;2;6 ddm1* mutants resemble the phenotypes of plants expressing centromeric histone CENH3 ‘tailswap’ GFP fusions, in which centromere function is impaired^31^. Therefore, we examined root tip anaphase cells in each of the genotypes for lagging chromosomes, an indication of impaired centromere function^31^. Remarkably, there was a strong chromosome lagging phenotype in *rdr1;2;6 ddm1* mutants, but not in the other genotypes (Fig. 2d; Table 1). This phenotype was ameliorated to some extent in each of the epigenetic suppressors of *rdr1;2;6 ddm1* (Table 1), although visible phenotypes returned in the next generation, consistent with the instability of these epialleles in a *ddm1* background. These data strongly suggested that centromere function was disrupted in *rdr1;2;6 ddm1* mutants, and was epigenetically inherited in the absence of RNAi (Fig. 1b).

**Table 1:** Mitotic chromosome mis-segregation in *rdr1;2;6 ddm1*

	Mis-segregation
WT	0%
*rdr1;2*	0%
*rdr1;2;6*	0%
*rdr1;2 ddm1*	0%
*rdr1;2;6 ddm1*	31%
*rdr1;2;6 ddm1 hp5* RNAi	3%
*tailswap cenh3*	19%
Episuppressor *3–75*	18%
*rdr1;2 ddm1 kyp*	13%
*rdr1;2 kyp*	0%

Chromosome mis-segregation was observed in root tip mitotic cells (*n* = 100) and the mis-segregation rate was calculated in the indicated strains. *tailswap cenh3*, a mutant defective in kinetochore function^31^, was used as a positive control.

## Retrotransposon small RNAs rescue defects in chromosome segregation

**Fig. 3:**
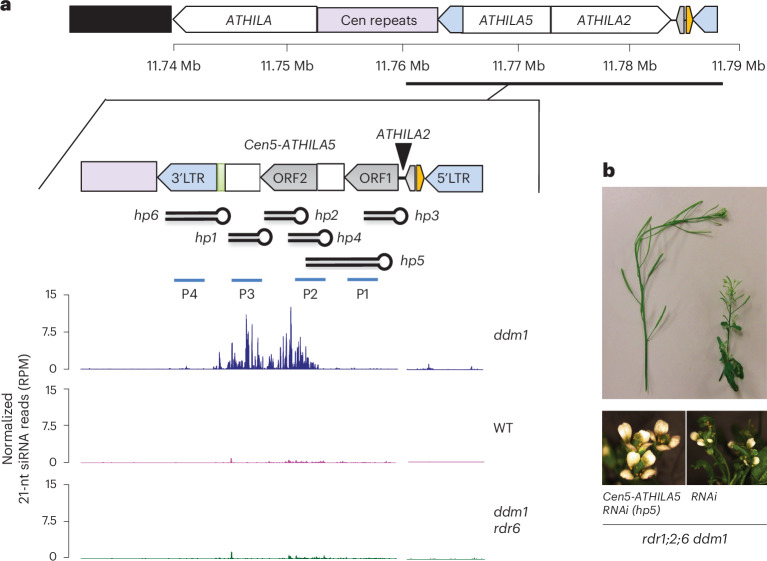
*Cen5-ATHILA5* hairpin small RNAs rescue *rdr1;2;6 ddm1* phenotypes. **a,** *Cen5-ATHILA5* is embedded in sequenced (purple) and unsequenced (black) centromeric repeats on chromosome 5 (TAIR10 genome assembly). It encodes two ORFs (grey arrows), short regions of homology to mitochondrial DNA (light green) and a tRNA gene (orange). Synthetic hairpins *hp1* through *hp6* and probes (P1 to P4) used for northern analysis (Extended Data Fig. 5) are shown (see Supplementary Table 2 for sequences). Genome browser tracks display 21-nt siRNA levels in indicated genotypes. Data from ref. ^18^. **b**, RNAi hairpin *hp5* strongly suppresses floral and fertility defects in *rdr1;2;6 ddm1*.

**Extended Data Fig. 4:**
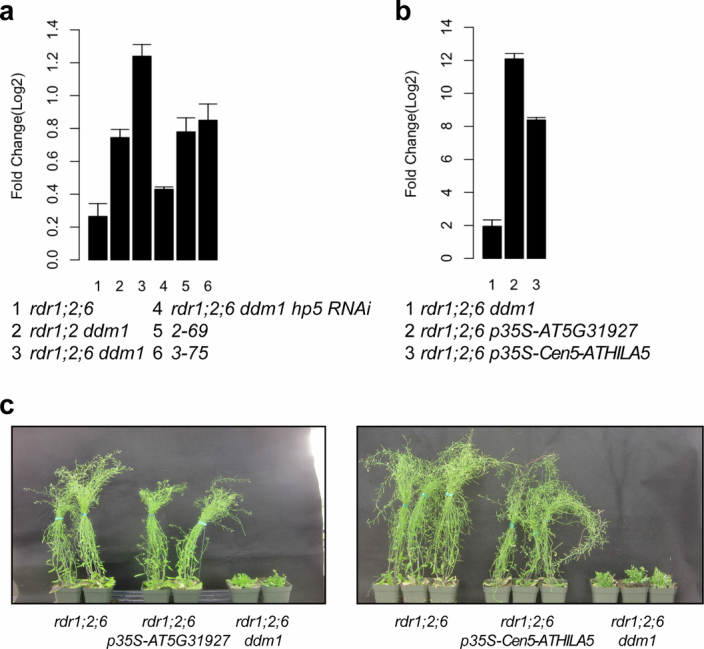
Overexpression of *Cen5-ATHILA5* does not cause developmental phenotypes in *rdr1;2;6* triple mutants. **a**, Expression levels of *Cen5-ATHILA5* in the indicated plants were analysed by RT-qPCR. Signals were normalized with *Cen5-ATHILA5* in WT. Bars represent standard error. **b**, RT-qPCR analysis for *Cen5-ATHILA5* in the plants overexpressing *AT5G31927* and *Cen5-ATHILA5*. Signals were normalized with *Cen5-ATHILA5* in WT. Bars represent standard error. **c**, Photos of 6-week-old *rdr1;2;6* plants overexpressing *AT5G31927* (left panel) and overexpressing *Cen5-ATHILA5* (right panel).

**Extended Data Fig. 5:**
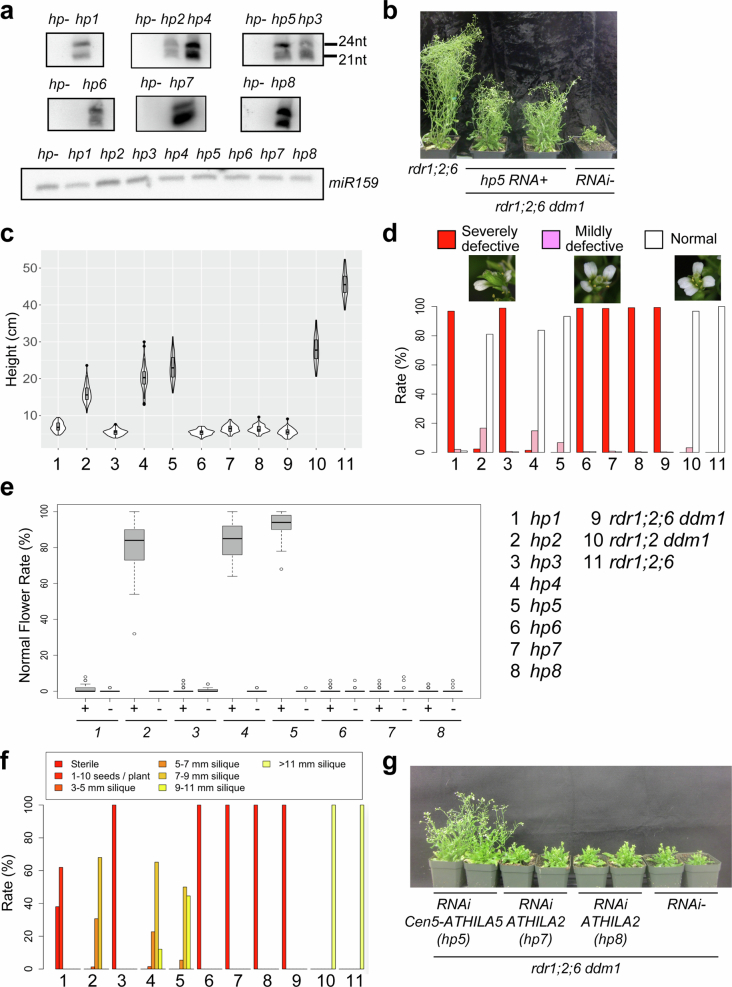
Hairpin suppressors of fertility and stature defects in *rdr1;2;6 ddm1.* **a**, *ATHILA5* and *ATHILA2* small RNAs were detected by Northern blot in the absence (*hp-*) or presence of hairpins shown in Fig. 3 (*hp1-6*: *Cen5-ATHILA5*; *hp7-8*: *ATHILA2*). As a loading control, abundantly expressed miR159 was detected on a different gel. **b**, Phenotypic suppression by *hp5* in *rdr1;2;6 ddm1*. **c**-**f**, The effects of *Cen5-ATHILA5* and *ATHILA2* hairpins on the *rdr1;2;6 ddm1* phenotypes (n = 96 plants): (**c**) height; (**d, e**) normal flowers and (**f**) fertility (silique length). The labelled numbers below the figures indicate the mutants shown next to (**e**). In (**c, e**), data are presented as median (black line), lower and upper quartiles (box) +/- 1.5 interquartile range (whiskers) and outliers (circles). **g**, 6-week-old *rdr1;2;6 ddm1* plants expressing *Cen5-ATHILA5* (*hp5*) or *ATHILA2* hairpins (*hp7* and *hp8*). See Supplementary Table 1 for source data. Source data

*Cen5-ATHILA5* encodes AT5G31927, which comprises two open reading frames (ORFs), the *GAG* gene (ORF1) and an *ATHILA* superfamily gene (ORF2), but ORF1 is interrupted by the integration of another retrotransposon, *ATHILA2*, potentially rendering it incompetent for further transposition (Fig. 3a). The expression level of *AT5G31927* is higher in *rdr1;2;6 ddm1* than in *rdr1;2 ddm1* or *rdr1;2;6*, and was silenced in the suppressor mutants (Extended Data Fig. 4a). We first hypothesized that proteins coded by *Cen5-ATHILA5* might be responsible for the mutant phenotype, but overexpression of the entire *Cen5-ATHILA5* element or ORF AT5G31927 did not cause any phenotype in *rdr1;2;6* mutant backgrounds (Extended Data Fig. 4b,c). Instead we considered the possibility that the loss of easiRNAs might be responsible, as *Cen5-ATHILA5* 21-nt easiRNAs accumulate in *ddm1*, but not in *ddm1 rdr6* (Fig. 3a)^18^. Simple overexpression of *Cen5-ATHILA5* would not be expected to restore easiRNAs in the absence of RNA-dependent RNA polymerase, so instead we introduced *Cen5-ATHILA5* hairpins into the *rdr1;2;6 ddm1* mutant as a source of double-stranded easiRNAs and siRNAs independent of RNA-dependent RNA polymerase (Fig. 3a). Hairpins corresponding to *ATHILA2* easiRNAs were also introduced as controls. These hairpins all generate *ATHILA* 21-nt and 24-nt small RNAs (Extended Data Fig. 5a).

**Fig. 4:**
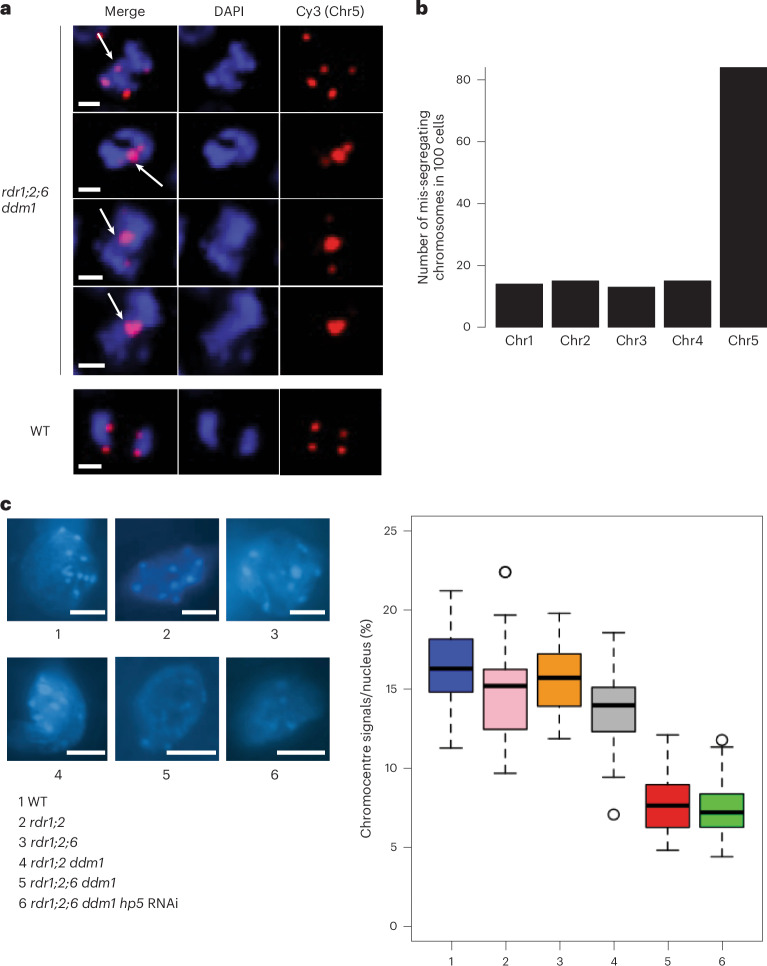
Chromosome mis-segregation in *rdr1;2;6 ddm1.* **a**, DNA FISH of root tip anaphase cells with Cy3-labelled DNA probes from chromosome 5 (red). Nuclei were counterstained with DAPI. Mis-segregating chromosomes are indicated with white arrows (scale bar, 2 µm; estimated from magnification). **b**, Numbers of mis-segregating chromosomes in *rdr1;2;6 ddm1* anaphase cells (*n* = 100 abnormal cells) as determined by FISH. **c**, Chromocentres were stained with DAPI (scale bar, 5 µm; estimated from magnification) and quantified signals (*n* = 30) illustrated by boxplots (right). Data are presented as mean values ± s.e.m.

Remarkably, hairpin-derived *Cen5*-*ATHILA5* small RNAs corresponding to both *ORF1* and *ORF2* (*hp5*) rescued infertility and many of the pleiotropic developmental defects of *rdr1;2;6 ddm1* mutants (Fig. 3b, Extended Data Fig. 5band Supplementary Table 1), while slightly milder suppression was observed for hairpins (*hp2,4*) targeting *ORF2* alone (Extended Data Fig. 5c and Supplementary Table 1). All three rescuing hairpins overlap with the easiRNA-accumulating region in *ddm1* mutants. This suppression was not observed when hairpins matching *ATHILA2* (including *hp1*, *hp7* and *hp8*) were introduced (Extended Data Fig. 5c–g). Most importantly, the high frequency of mitotic chromosome mis-segregation in the *rdr1;2;6 ddm1* mutant was also greatly reduced in the *Cen5-ATHILA5* hairpin *hp5* suppressor (Table 1). Because *Cen5-ATHILA5* is embedded within the 178 bp centromeric satellite repeats of chromosome 5 (Fig. 3a), the mis-segregating chromosomes observed in the *rdr1;2;6 ddm1* mutant should correspond to chromosome 5. To test this hypothesis, we performed DNA fluorescence in situ hybridization (FISH) using mitotic cells from root tips in the *rdr1;2;6 ddm1* mutant. The proportion of mis-segregating chromosome 5 was calculated by co-localization of Cy3 probe signals with the observed chromosomal mis-segregation. Of the mis-segregating chromosomes, 84% correspond to chromosome 5, with lower proportions for the other chromosomes (Fig. 4a,b). Thus, artificial siRNAs derived from the *Cen5-ATHILA5* retrotransposon hairpins are sufficient to restore accurate chromosome segregation.

## Retrotransposon small RNAs promote DNA methylation and H3K9me2

Recently, the centromeric sequences of Col-0 have been assembled with single-molecule long-read sequencing technology^32^. Unexpectedly, multiple copies of full-length *ATHILA5* and other *ATHILA* retrotransposons were specifically found embedded into the CENH3-containing centromeric repeats of centromere 5. Single molecule long read sequencing and assembly of other *Arabidopsis* accessions have since revealed that waves of *ATHILA5* retrotransposons have recently and specifically disrupted centromeres 4 and 5 in several sympatric accessions of *Arabidopsis* from Europe^33^. Invasion seems to have disrupted homogenization of satellite repeats, suggesting these insertions may interfere with recombination mechanisms, such as break-induced replication and repair^33^.

**Fig. 5:**
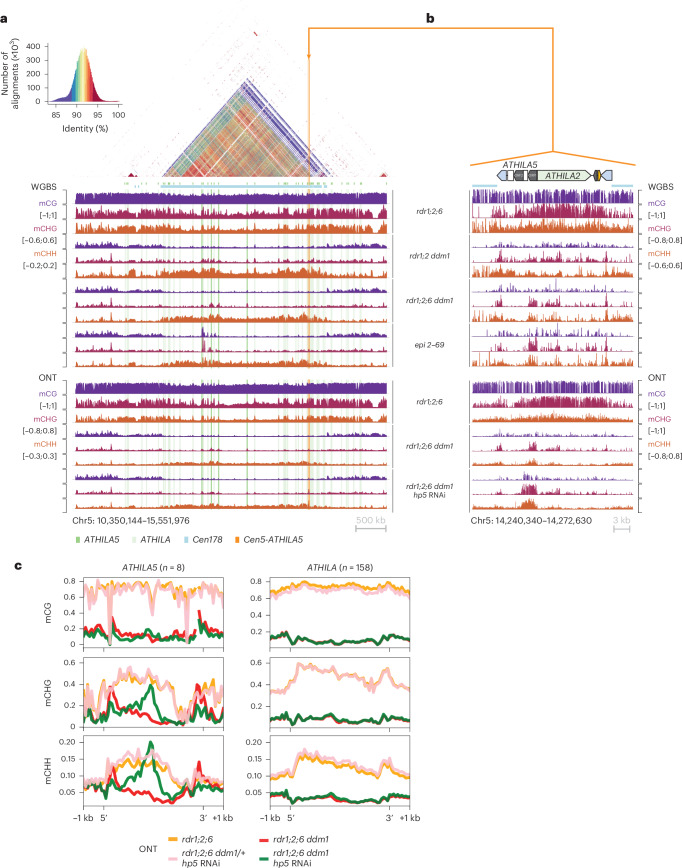
easiRNAs restore non-CG DNA methylation to *ATHILA5* elements on chromosome 5. **a**,**b**, Browser screenshots of the centromeric region of chromosome 5 (**a**) and *Cen5-ATHILA5* (**b**), showing DNA methylation (mC/C in each sequence context) from WGBS, and DNA methylation called from long reads (ONT). The loss of DNA methylation in the *rdr1;2;6 ddm1* mutant is partially recovered at *Cen5-ATHILA5* with expression of the RNAi hairpin *hp5*. Values are averaged in windows of 5 kb (**a**) or 10 bp (**b**). The dotplot (**a**) reveals high identity between the cen178 repeats (light blue bar), with interspersed *ATHILA* elements (light green columns) and *ATHILA5* elements (dark green columns). The *CEN5-ATHILA5* used to design the RNAi hairpin is also shown (orange column, inset). **c**, Metaplots of DNA methylation (from ONT) over *ATHILA5* (mean, *n* = 8, not including hp5 containing *CEN5-ATHILA5*) and all other *ATHILA* (mean, *n* = 158) in the genome. Levels of non-CG methylation in the *rdr1;2;6 ddm1* mutant are recovered specifically at *ATHILA5* elements when the hp5 is present.

Mapping of our WGBS data revealed that satellite repeats lost CG and CHG methylation in *ddm1* mutant combinations, but retained CHH methylation as expected (Fig. 5a). However, the *ATHILA* elements in centromere 5 retained some CHG and especially CHH methylation in *rdr1;2 ddm1*, but substantially less in *rdr1;2;6 ddm1* (Fig. 5a). Furthermore, WGBS data from a heritable epigenetic suppressor of *rdr1;2;6 ddm1* (suppressor *2–69*, Fig. 2a,b) also revealed ectopic DNA methylation at *ATHILA* elements, but in all sequence contexts (Fig. 5a,b). To detect methylation at cytosine residues unambiguously in highly repetitive regions, we performed single-molecule long-read genome sequencing using Oxford Nanopore Technologies (ONT), and profiled methyl cytosine using base-calling protocols (Methods). We compared methylation patterns in *rdr1;2;6 ddm1*, and in *rdr1;2;6 ddm1/*+ siblings, with and without the *Cen5-ATHILA5* (*hp5)* hairpin suppressor (Fig. 5a,b). On metaplots of *ATHILA5* elements, but not other *ATHILA* elements, DNA methylation was specifically restored precisely in the region defined by the hairpin (Fig. 5c). DNA methylation was only restored in the CHG and CHH contexts, and not in the CG context, consistent with it being induced by RNAi^34^ (Fig. 5c).

**Fig. 6:**
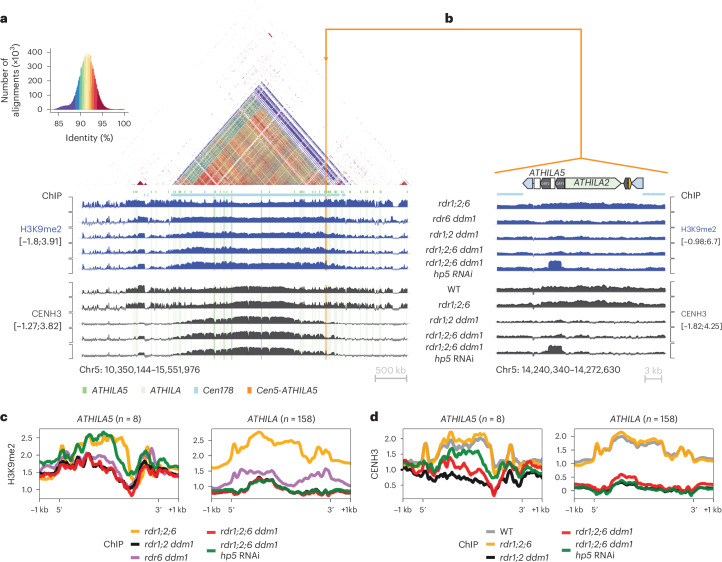
EasiRNAs restore H3K9me2 and CENH3 levels to *ATHILA5* elements on chromosome 5. **a**,**b**, Browser screenshots of the centromeric region of chromosome 5 (**a**) and *Cen5-ATHILA5* (**b**), showing H3K9me2 tracks (log_2_[IP/input]) and CENH3 tracks (log_2_[IP/input]). Similar to DNA methylation (Fig. 5), the loss of H3K9me2 and CENH3 in the *rdr1;2;6 ddm1* mutant is partially recovered at the *Cen5-ATHILA5* with expression of the RNAi hairpin *hp5*. Values are averaged in windows of 5 kb (**a**) or 10 bp (**b**). The dotplot (**a**) reveals high identity between the cen178 repeats (light blue bar), with interspersed *ATHILA* elements (light green columns) and *ATHILA5* elements (dark green columns). The *CEN5-ATHILA5* used to design the RNAi hairpin is also shown (orange column, inset). **c**,**d**, Metaplots of H3K9me2 (**c**) and CENH3 (**d**) over *ATHILA5* (mean, *n* = 8, not including *hp5* containing *CEN5-ATHILA5*) and all other *ATHILA* (mean, *n* = 158) in the genome. Levels of H3K9me2 and CENH3 in the *rdr1;2;6 ddm1* mutant are recovered specifically at *ATHILA5* elements when the *hp5* is present.

**Extended Data Fig. 6:**
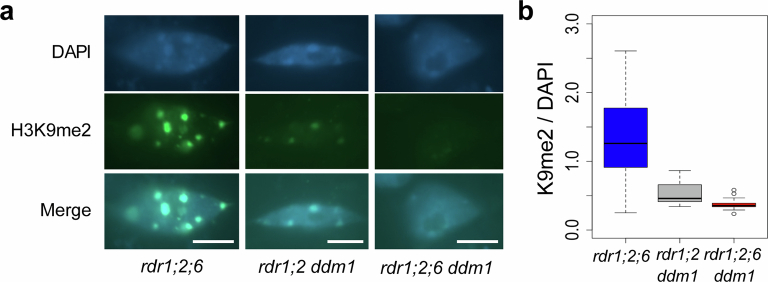
Reduced H3K9me2 at chromocentres in *rdr1 rdr2 rdr6 ddm1.* **a**, Immunofluorescence of H3K9me2 in mature leaf nuclei was observed in the indicated genotypes, and nuclei were counterstained with DAPI (top panels). Scale bar = 5 µm, estimated from magnification. **b**, Ratios of H3K9me2 to DAPI in chromocentres (n = 30) of each genotype. Data are presented as median (black line), lower and upper quartiles (box) +/- 1.5 interquartile range (whiskers) and outliers (open circles).

**Extended Data Fig. 7:**
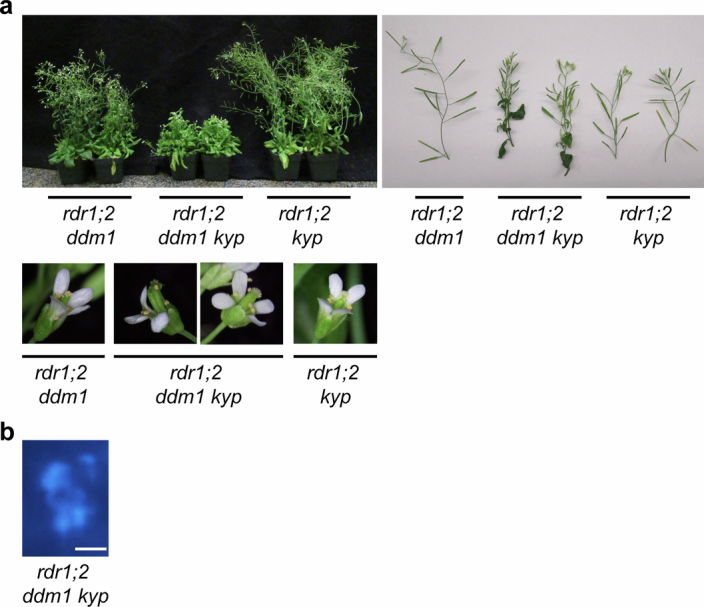
Developmental phenotypes and chromosome mis-segregation defects in *rdr1;2 ddm1 kyp.* **a**, Aggravated phenotypes of *rdr1;2 ddm1 kyp* compared to *rdr1;2 ddm1* and *rdr1;2 kyp*. *rdr1;2 ddm1 kyp* plants exhibited partially deformed flowers (abnormal; 71%, normal; 29%) and reduced fertility. **b**, Mis-segregating chromosomes during anaphase in *rdr1;2 ddm1 kyp*. Scale bar = 2 µm, estimated from magnification.

CHG and CHH DNA methylation depend on histone lysine-9 di-methylation (H3K9me2) via the chromodomain DNA methyltransferases CHROMOMETHYLTRANSFERASE2 (CMT2) and 3 (CMT3). In *S.* *pombe*, RNAi mutants lose H3K9me2 and suffer from severe chromosome mis-segregation due to loss of sister chromatid cohesion^9,10^. In *Arabidopsis ddm1* mutants, *RDR6*-dependent easiRNAs derived from pericentromeric transposons also induce H3K9me2 (ref. ^35^), and we postulated that they might have a role in centromeric organization. We observed that chromocentres in *rdr1;2;6 ddm1* were greatly diminished when compared with those in *rdr1;2 ddm1* or *rdr1;2;6* mutants (Fig. 4c), suggesting that *RDR6* activity, specifically in the absence of DNA methylation, is required for pericentromeric heterochromatin condensation. We then investigated the effect of *rdr6* on histone modification by comparing *rdr1;2;6* and *rdr1;2;6 ddm1* mutants, with and without the addition of *hp5*. We performed chromatin immunoprecipitation sequencing (ChIP-seq)^36^ and found that H3K9me2 is highly enriched in multiple families of *ATHILA* elements in WT, but reduced in *rdr1;2 ddm1* and *rdr1;2;6 ddm1* (Fig. 6a), a result which was confirmed by immunofluorescence (Extended Data Fig. 6). However, this decrease in H3K9me2 was almost fully restored by the *Cen5-ATHILA5* hairpin suppressor, along with chromosome segregation (Fig. 6b,c). Thus, easiRNAs ensure pericentromeric H3K9me2 at *ATHILA5* elements in centromere 5. Severely diminished chromocentres, sterile and developmental phenotypes are also observed when *Arabidopsis* loses both histone H3K9 and DNA methylation^37^. We further tested this idea by making mutant combinations with *KRYPTONITE* (*AT5G13960*), one of several H3K9 methyltransferases in *Arabidopsis*^34,38^. We found that vegetative phenotypes of *rdr1;2 ddm1 kyp* mutants resembled *rdr1;2;6 ddm1* mutants, including defects in chromosome segregation, although floral defects were less severe (Extended Data Fig. 7).

DDM1 has recently been shown to be required for the replacement of H3.3 by H3.1 (ref. ^39^), and we speculated that it might also impact the distribution of CENH3, an H3.3 variant. We performed ChIP-seq using an antibody against CENH3, and found that CENH3 was localized, as expected, throughout the centromeric satellite region in WT and in *rdr1;2;6* mutants, and extended at lower levels into the flanking pericentromeric regions^32^. Unlike H3.3, however, CENH3 was lost from pericentomeric domains in *rdr1;2 ddm1* mutants, and in all the other genotypes tested (Fig. 6a), as well as from *ATHILA* elements embedded within the repeats (Fig. 6d). This distribution closely resembled the distribution of H3K9me2 (Fig. 6a–c), which was similarly lost from the pericentromeric domain and from transposons in *ddm1* mutants. However, both H3K9me2 and CENH3 were retained at high levels by satellite repeats. Intriguingly, introduction of the *hp5* hairpin that generated large numbers of 21–24-nt small RNA corresponding to *Cen5-ATHILA5*, resulted in restoration of both H3K9me2 and CENH3 to related *ATHILA5* elements embedded within the satellite repeats, and especially to the *Cen5-ATHILA5* element itself (Fig. 6b–d).

**Fig. 7:**
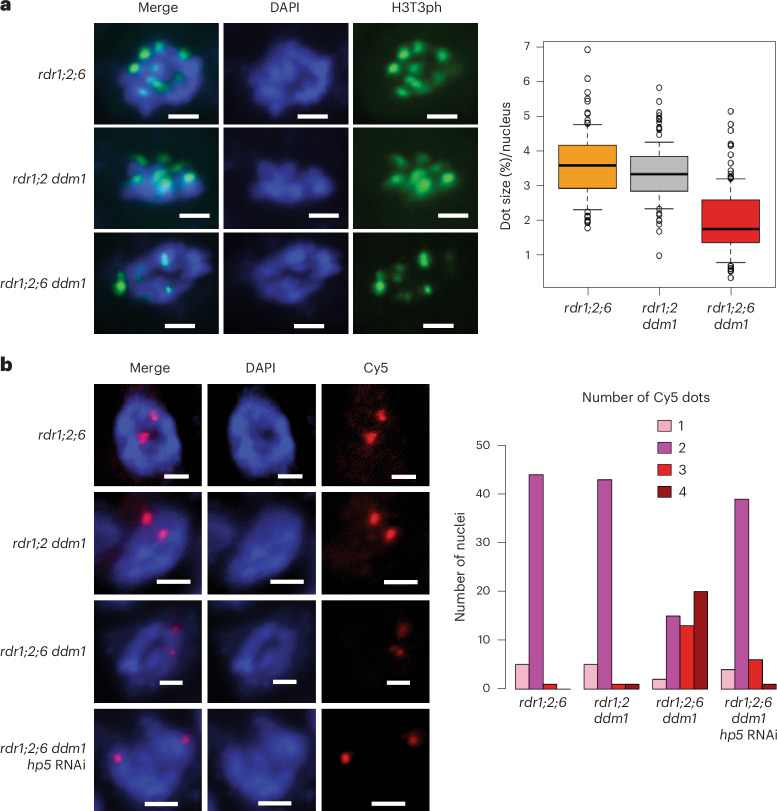
Defective sister chromatid cohesion is restored by easiRNAs in *rdr1;2;6 ddm1.* **a**, Immunofluorescence for H3T3ph in root tip cells. Left panels exhibit mitotic prophase cells showing H3T3ph signals. Cells were counterstained with DAPI. In prophase cells, condensed DAPI dots are dispersed in the nucleus (scale bar, 2 µm). H3T3ph dot sizes were calculated based on the nucleus size (right panel). In total, 100 dots from 20 nuclei were analysed. Data are presented as median (black line), lower and upper quartiles (box) ± 0.5 × interquartile range (whiskers) and outliers (open circles). **b**, DNA FISH in mitotic prophase cells with Cy5-labelled probes designed near the pericentromeric region of chromosome 5 (left panels, scale bar; 2 µm). The right panel shows the number of Cy5 dots (1–4) in each nucleus. In total, 50 nuclei were analysed for each mutant.

Pericentromeric heterochromatin near the kinetochore includes the inner centromere, which connects sister kinetochores before anaphase through chromosome cohesion. In mammals and yeast, the inner centromere functions as a scaffold to recruit factors important for chromosome segregation such as Aurora kinase, shugoshin, cohesin and condensin^40^. Although the *Arabidopsis* inner centromere has not been well characterized, cohesin and condensin are enriched in the pericentromere^41,42^, and mutation of these factors affects pericentromeric architecture and chromosome mis-segregation^42,43^. Further, histone residues H3S10 and H3T3 are highly phosphorylated specifically at the pericentromeric region during mitosis^44^, and the activity of Aurora kinase is essential for chromosome segregation^45,46^. We examined H3T3 phosphorylation by antibody staining, and could clearly detect phosphorylation at chromocentres, which were smaller in *rdr1;2;6 ddm1* than *rdr1;2 ddm1* as expected (Fig. 7a). Next, we used DNA FISH of chromosome 5 to examine cohesion in the mutants. By counting the number of fluorescent foci, we could assess whether cohesion was normal at mitosis (two foci), or reduced (three or four foci). We found that cohesion was dramatically lost in *rdr1;2;6 ddm1* mutants, but fully restored by the *Cen5-ATHILA5* (*hp5*) hairpin (Fig. 7b).

## Discussion

**Extended Data Fig. 8:**
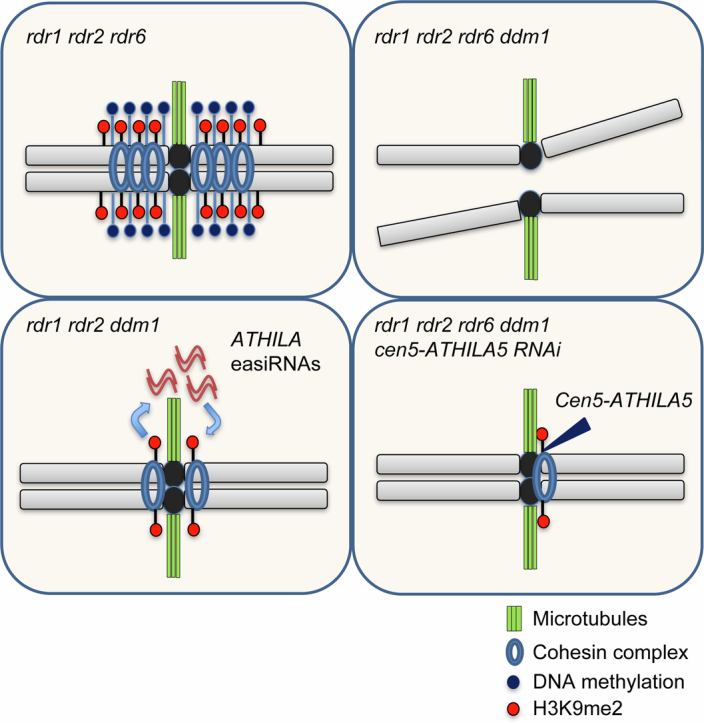
A model for the regulation of pericentromeric sister chromatid cohesion by DNA methylation and small RNAs. The Arabidopsis pericentromere is maintained by DNA methylation and H3K9 methylation, and is essential for sister chromatid cohesion. When DNA methylation is lost, plants produce RDR6-dependent easiRNAs from *ATHILA* family retrotransposons, enriching H3K9 methylation at pericentromeric *ATHILAs*. Additional loss of easiRNAs causes impaired sister chromatid cohesion and severe mis-segregation of chromosome 5. The sterility, and the sister chromatid cohesion defect of chromosome 5 can be rescued by artificial small RNAs targeting retrotransposon *ATHILA5*, which re-establishes H3K9-methylated heterochromatin.

**Extended Data Fig. 9:**
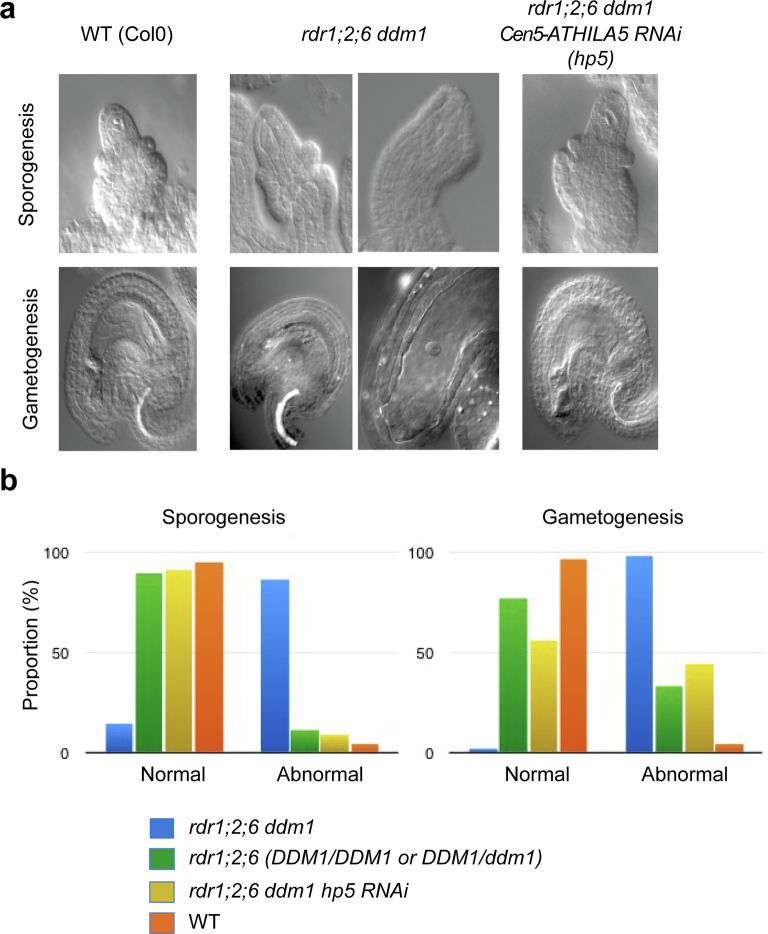
Defective female sporogenesis and gametogenesis in *rdr1;2;6 ddm1.* **a**, Female sporogenesis and gametogenesis were analysed by whole-mount ovule clearing in the indicated strains. In the quadruple mutant, presence of multiple megaspore mother cells (mmc) was noted in 32% of ovules during sporogenesis. Lack of a clear mmc was observed in 12% of ovules scored. Conspicuous absence of a gametophyte, or incomplete gametophytes were found in most (>76% of ovules), a phenotype which was partially rescued in the suppressor lines. **b**, Quantification of the phenotypes in the indicated strains.

We have demonstrated that *RDR6*-dependent 21-nt easiRNAs compensate for loss of DNA methylation by promoting pericentromeric chromatin condensation and proper mitotic chromosome segregation (Extended Data Fig. 8). We did not examine meiotic chromosome segregation because of the difficulty of identifying meiotic cells in *rdr1;2;6 ddm1* quadruple mutants, and it is likely that developmental defects may account for their near-complete infertility (Extended Data Fig. 9). We observed that *RDR6*-dependent 21-nt easiRNAs facilitate histone H3K9 methylation in the absence of DDM1, and are required for chromosome segregation and normal development. Importantly, the phenotypic defects in *rdr1;2;6 ddm1* were rescued by restoring small RNAs and histone H3K9 methylation via hairpin precursors that match *Cen5-ATHILA5*, a Ty3/gypsy class retrotransposon family embedded specifically within *Cen5* centromeric repeats. Similar hairpin precursors induce H3K9me2 and non-CG DNA methylation in *Arabidopsis*^34,47^. However, we did not observe any difference in H3K9me2 levels between *rdr6 ddm1*, *rdr1;2 ddm1* and *rdr1;2;6 ddm1* mutants (Fig. 6c), despite having differing levels of chromosome segregation (Fig. 7). As *rdr1;2 kyp ddm1* mutants resemble *rdr1;2;6 ddm1* mutants in this respect, we speculate that an additional histone modification is likely guided by 24-nt siRNAs, mediated by *RDR2*, and that both modifications are likely required for cohesion.

In the fission yeast *S.* *pombe*, which lacks DNA methylation, RNAi promotes sister chromatid cohesion by recruiting cohesin to pericentromeric heterochromatin and allowing proper chromosome segregation^9,10^. In mouse, *dicer* mutant ES cells also have strong centromeric segregation defects, and these can be rescued by mutations in conserved transcription factors that also rescue *dcr1* mutants in fission yeast^48^. In humans, patients with ICF syndrome (immunodeficiency, centromere function and facial abnormalities) have mutations in *HELLS*, the *DDM1* orthologue, or in other genes required for DNA methylation, and HEK293 cells mutant for these genes have defects in chromosome segregation and DNA methylation^49^. This suggests that mammalian cells require both RNAi and DNA methylation for centromere function. In *Arabidopsis*, we show that chromosome segregation can be maintained by either RNAi or DNA methylation alone, so that only mutants that lose both have segregation defects. In each species, including mammals and plants^11,48^, these effects are likely mediated by centromeric transcription which is silenced by histone H3K9 methylation, promoting cohesion. Humans lack RNA-dependent RNA polymerase, which amplifies siRNAs in yeast and *Arabidopsis*, and loss of *HELLS*^DDM1^ alone leads to immune and centromere defects^50^. Hence, siRNAs targeted to centromeric repeats may offer a potential therapeutic avenue for ICF syndrome.

While segregation of all five chromosomes was defective in *rdr1;2;6 ddm1*, mis-segregation of chromosome 5 had the largest phenotypic contribution, and co-segregated epigenetically with the local loss of DNA methylation in this interval, strongly supporting the idea that centromere function is an epigenetic property^51^. We note that trisomics of chromosome 5, among all the *Arabidopsis* trisomics, exhibit the most severe defects in fertility^52^, which might explain why fertility defects mapped to this centromere in particular. The *Arabidopsis* inner centromere comprises tens of thousands of 178 bp repeats, but Col-0 chromosome 5 stands out in having been recently invaded by *ATHILA* retrotransposons, notably by *ATHILA5* (ref. ^32^). It has previously been reported that a subset of centromeric satellite repeats are transcribed but post-transcriptionally silenced by DCL1, which triggers easiRNAs^11,17^. Sequence comparison indicates that these repeats bind CENH3 (ref. ^53^). Another subset of satellite repeats is transcriptionally silenced by DDM1, which prevents transcription from embedded *ATHILA* retrotransposons and their derivatives^11^. Both classes are associated with DNA methylation and H3K9me2 (ref. ^32^). Thus, in addition to centromere disruption, insertion of centromeric *ATHILA* retrotransposons silences transcription from centromeric repeats by a combination of DNA methylation, RNAi and H3K9me2. Centromere transcription and silencing is thought to be required for both cohesion and for loading of CENH3 (ref. ^54^), and consistently, CENH3 is lost from *ATHILA* elements and from pericentromeric regions in *rdr1;2 ddm1* mutants along with H3K9me2, including from outer satellite repeats (Fig. 6a). Further, CENH3 and H3K9me2 are ectopically acquired at *ATHILA5* elements when they are targeted by hairpin small RNA. However, both CENH3 and H3K9me2 are retained at normal levels over inner satellite repeats, which are therefore still capable of forming a kinetochore. These observations are consistent with a cohesion defect, rather than a kinetochore defect, being responsible for chromosome mis-segregation in *rdr1;2;6 ddm1* when DNA methylation and RNAi are simultaneously compromised.

The loss of centromere function that has recently been disrupted and silenced, suggests that retrotransposon invasion makes centromeres dependent on these elements, especially when they are epigenetically compromised. While mutants in *ddm1* have not been found among *Arabidopsis* accessions in the wild, large hypomethylated regions up to 5 Mb have been found, and have similar phenotypic effects as *ddm1* (ref. ^29^). Such regions likely arise transiently in populations, but convey a fitness benefit in subsequent generations. Thus, centromeres can become ‘addicted’ to invading retrotransposons via RNAi and silencing^55^, an apparently successful strategy for retrotransposon survival in *Arabidopsis*^33^. Similar strategies may have been deployed by transposons in maize^56^ and in a close fission yeast relative^57^ whose centromeres have also been recently invaded by retrotransposons.

## Methods

### Plant strains, preparation of DNA and RNA, primers

*ddm1-1* mutants with mutations of RNA-dependent RNA polymerase genes (*rdr1* (SALK_112300) *rdr2* (SALK_059661) *rdr6-11*) were generated in the previous study^22^. *tailswap cenh3* is a kind gift from S. W. L. Chan. The *kyp-4* (SALK_044606) mutant was used. DNA was extracted from leaves of 4-week-old plants by Nucleon Phytopure (GE Healthcare) and total RNA was extracted from 3-week-old plants by RNeasy (QIAGEN) or Direct-zol (ZYMO RESEARCH). All primers and oligonucleotides used in this study are listed in Supplementary Table 2.

### Construction of epi-recombinant lines

*rdr1 rdr2 ddm1* was crossed to *rdr1 rdr2 RDR6/rdr6* to obtain *rdr1 rdr2 DDM1/ddm1 RDR6/rdr6* plants in F1. The F1 *rdr1 rdr2 DDM1/ddm1 RDR6/rdr6* plants were crossed to *rdr1 rdr2 RDR6/rdr6*. *rdr1 rdr2 rdr6/rdr6 DDM1/DDM1* were selected in F2 and DNA were extracted from the rosette leaves of 4-week-old plants individually (10 fertile and 10 sterile plants), followed by WGBS as described below. DNA methylation levels in all three cytosine contexts (CG, CHG and CHH) in 100 kb fixed windows were calculated for each sample, and the average DNA methylation levels for the fertile and sterile groups were compared.

### Hairpin small RNA complementation

The 35S promoter and nos terminator were cloned into pPZP2H (ref. ^58^) to make an expression vector (p35S-pPZP2H) at KpnI-ApaI and XbaI-SacI site, respectively. A partial *Cen5-ATHILA5* element and its inverted form separated with *GUS* spacer were amplified by PCR (T8H11 BAC DNA and *Escherichia* *coli* genomic DNA were used as templates to amplify *Cen5-ATHILA5* and *GUS* fragments) and cloned into p35S-pPZP2H, resulting in inverted repeats of *Cen5-ATHILA5* in the expression vector. After transformation using *Agrobacterium tumefacien*s, *DDM1/ddm1* T1 transformants were selected with hygromycin resistance, and the T2 plants were grown without hygromycin selection for each hairpin. 96 *ddm1/ddm1* T2 plants from 6 independent T1 lines (16 × 6) were isolated by genotyping and phenotyping was performed, followed by confirmation of the hairpin construct insertion by PCR. Of the 96 examined plants, the number of plants which had the hairpin construct were: 71 (*hp1*), 75 (*hp2*), 76 (*hp3*), 66 (*hp4*), 74 (*hp5*), 67 (*hp6*), 72 (*hp7*), 74 (*hp8*). For phenotyping, 9-week-old plants were used for assessing height and fertility, and 7-week-old plants for the flower phenotype. 50 flowers were analysed for each plant, and overall fertility was estimated based on seed availability (sterile; 1–10 seeds per plant) and primary developing silique length 3–5 mm (approximately 1–5 seeds per silique); 5–7 mm (approximately 5–10 seeds per silique); 7–9 mm (approximately 10–15 seeds per silique); 9–11 mm (approximately 15–20 seeds per silique); >11 mm (more than 20 seeds per silique)). Note that the *ddm1/ddm1* plants that segregated in T2 without hairpins were all sterile and did not show suppression for the height and flower phenotypes, and *hp5* suppressors were fertile at least for three generations after the plants become *ddm1*/*ddm1*, although the fertility was reduced more in later generations. Because *hp5* showed the strongest suppression, we subsequently isolated a T3 homozygous *hp5* insertion line with the heterozygous *DDM1* mutation, and used T3 *ddm1/ddm1* hp5 suppressors for RT-PCR, ChIP-seq and cytogenetics. For construction of *Cen5-ATHILA5* overexpressing plants, the *Cen5-ATHILA5* element or its ORF AT5G31927 were cloned into pMDC45 expression vector at the KpnI-SpeI site and the vectors were transformed into *rdr1;2;6* and approximately 16 T1 plants were phenotyped. The images and qRT-PCR data for the overexpressing lines were taken in selfed T2 plants.

### *rdr1 rdr2 rdr6 ddm1* suppressor analysis

Seeds of *rdr1 rdr2 rdr6 DDM1/ddm1* were mutagenized with EMS and *DDM1/ddm1* plants (approximately *n* = 500) were selected by genotyping of the M1 generation. In M2, *rdr1;2;6 ddm1* plants with rescued sterility and floral defects were isolated by checking approximately 3,000 M2 plants showing curly leaf and short stature phenotypes, followed by confirmation of the *ddm1* homozygous mutation by genotyping. EMS-induced SNPs in *rdr1;2;6 ddm1* suppressors were identified by whole-genome sequencing (Illumina Hiseq2000). Suppressors’ parental M2 seeds (*rdr1 rdr2 rdr6 DDM1/ddm1* bearing the heterozygous suppressor mutation) were planted to segregate suppressors and non-suppressors in the same M3 progeny, allowing us to perform CAPS analysis. In total, 15 suppressors and 45 non-suppressors were analysed for each suppressor. SNPs in the centromeric region of chromosome 5 and restriction enzymes used for CAPS analysis are as follows: 10483242 G to A and PacI (*2–69*), 11316097 G to A and Hpy188I (*2–20*), 13349168 G to A and HhaI (*3–72*), 13818243 C to T and AflII (*3–75*).

### RNA analysis

Total RNA (10 μg) was used for electrophoresis on 15% Acrylamide Urea-TBE gel. Separated RNA was transferred onto Hybond-NX membrane (GE Healthcare) and the membrane was crosslinked with EDC (1-ethyl-3-(3-dimethylaminopropyl)carbodiimide). RNA probes for detecting *ATHILA*-derived small RNAs were generated in vitro as recommended by the manufacturer (Ambion). To prepare a probe for miR159 detection, its complementary oligo nucleotide DNA was labelled with radioactive phosphate (Perkin Elmer). For quantitative RT-PCR 1 μg of total RNA was treated with 5 Units of DNase I (Takara) and cDNA synthesized with SuperScriptIII (Life Technologies) was used for the subsequent qPCR analysis.

### Whole-genome bisulfite sequencing

WGBS was performed as described previously^18^. Briefly, 1 µg of genomic DNA was sheared with Covaris S220 and purified with QIAquick PCR purification kit (QIAGEN 28106). DNA libraries were constructed with DNA library preparation kit (NEB6040) using cytosine-methylated adaptors (NEXTflex bisulfite-seq barcodes-12, Bioo Scientific 511912). The libraries were treated with sodium bisulphite using EZ DNA Methylation-Gold Kit (Zymo Research D5005) according to the protocol provided by the manufacturer, followed by PCR amplification with Expand High Fidelity PLUS PCR system (Roche 03300242001). Libraries were sequenced with Hiseq 2000 or Hiseq 2500 in a paired-end 101 bp protocol. Reads were mapped using Bismark^59^. Initially, hypermethylated regions common to all four suppressors were identified by genome browsing, but robustness was then assessed by DMR analysis. For analysing DMRs, total DNA methylation levels in 300 bp were calculated by summing all CG/CHG/CHH methylation levels. Of the regions retaining less than 40% of DNA methylation levels in *rdr1;2;6 ddm1* compared with those in *rdr1;2;6*, the regions recovering more than 60% in suppressors were sorted as hyper-methylated DMRs in suppressors.

### Chromatin immunoprecipitation sequencing

ChIP-seq were perfomed as described previously^60^, with some modifications. Frozen two-week-old seedlings (0.5 g) were ground under liquid nitrogen, and the ground tissues were crosslinked with 12.5 ml of formaldehyde solution (1% formaldehyde, 10 mM HEPES pH 7.6, 1 M sucrose, 5 mM KCl, 5 mM MgCl_2_, 5 mM EDTA, 0.6% Triton-X100, 0.1% 2-mercaptoethanol, 1× complete protease inhibitor (Sigma), pH 8.0) for 10 minutes at room temperature. Crosslinking reaction was quenched by adding 0.85 ml of 2 M glycine and samples were incubated for 5 minutes at room temperature. The tissues were further broken up with a dounce homogenizer, followed by nuclear pellet isolation and resuspension in 150 µl of SDS Lysis buffer (50 mM Tris-HCl pH 7.8, 1% SDS, 10 mM EDTA pH 8.0). The samples were incubated at 4 °C for 10 minutes and diluted with 1.85 ml of buffer 1 (50 mM HEPES/KOH (pH 7.6), 140 mM NaCl, 1 mM EDTA, 1% Triton X-100, 0.1% Na-Deoxycholate, 1× complete protease inhibitor). Bioruptor UCD-200 (Diagenode) was used to obtain 250–500 bp sheared chromatin. After centrifugation at 15,000 rpm and 4 °C for 10 minutes, the supernatant was used for immunoprecipitation. Primary antibody (4 µl) against H3K9me2 (Abcam, ab1220) or against CENH3 (gift of S. Henikoff, Fred Hutchinson Cancer Research Center, Seattle, WA, USA^61^) were used for immunoprecipitation. Input and washed immunoprecipitated samples resuspended in TE buffer were treated with 0.1 mg ml^–1^ RNase A at 37 °C for 30 minutes and with 0.25 mg ml^–1^ Proteinase K and 0.25% SDS at 42 °C for 1 hr. Samples were then reverse-crosslinked at 65 °C overnight, followed by purification with QIAquick PCR purification kit (QIAGEN 28106). ChIP-seq libraries were made by NEB Next Ultra II DNA Library Prep Kit (E7645) and NEBNext Multiplex Oligos for Illumina (E7335) following the manufacturer’s instructions. Libraries were sequenced with Nextseq 500 paired-end 76 bp. ChIP-seq libraries were made by NEB Next Ultra II DNA Library Prep Kit. FASTQ files were trimmed with cutadapt^62^ and mapped to Col-CEN v1.2 ref. ^32^ with Bowtie2 (ref. ^63^). Mapped files were processed with SAMtools^64^ and DeepTools^65^ to generate browser tracks. Duplicate reads were kept as H3K9me2 and CENH3 are enriched at repetitive or multi-copy elements, but conclusions were unchanged regardless of removing duplicate reads.

### Long-read DNA sequencing (ONT) and methylation base-calling

DNA was extracted from approximately 100 mg of rosette leave from *rdr1;2;6 ddm1, rdr1;2;6 Cen5-ATHILA5 ddm1* (*hp5*) and their corresponding DDM1 wild-type siblings with the DNeasy Plant Pro Kit (Qiagen). From each sample, 600 ng to 1 µg of purified DNA was taken as input for ligation library preparation with the Native Barcoding Kit 24 v14 (ONT - SQK-NBD114.24). A 35 ng portion of the multiplexed library was sequenced on an R10.4.1 PromethION flow cell. Standard and modified (5mC) base calling was carried out with dorado v0.5.0 (ONT) using the dna_r10.4.1_e8.2_400bps_sup@v4.1.0 and res_dna_r10.4.1_e8.2_400bps_sup@v4.0.1_5mC@v2 models. Reads were aligned to the Col-CEN v1.2 (ref. ^32^) with minimap2 v2.26-r1175 (ref. ^66^), and consensus methylation calls were produced at each cytosine with modkit v0.2.3 (ONT) “pileup–combine-mods”. Calls at positions with at least three reads were retained and the remaining calls were split by cytosine context (CpG, CHG, CHH) using modkit motif-bed and bedtools v2.31.0 (ref. ^67^) intersect. Methylation ratios at each position were scaled to the [0-1] interval, and ratios on the (-) reference strand were multiplied by –1 before conversion to BigWig format with UCSC tools^68^.

### Cytogenetics

Seedlings (1 week old) were soaked in 1 mg ml^–1^ of DAPI solution containing 0.1% Triton X-100 for 10 min at room temperature. DAPI-stained chromosomes were analysed with ZEISS microscopy. To calculate proportion of chromocentre signals in nucleus, DAPI signals from 30 chromocentres were analysed by Image J v1.52 (ref. ^69^). DNA FISH was performed as described previously^41^. For preparing probes, two contiguous BAC clones were used to detect each chromosome: T1F9 F11P17 (Chr 1), T2G17 F11A3 (Chr 2), MIPN9 MIMB12 (Chr 3), F6I7 F13M23 (Chr 4), MINC6 K19P17 (Chr 5, Fig. 4a) and T1G16 T1N24 (Chr 5, Fig. 7b). Probes were labelled by nick translation with Cy3-dUTP or Cy5-dUTP as recommended by the supplier (Promokine). Fluorescent signals were analysed by confocal microscopy. Immunofluorescence experiments were performed as described previously^36^. The antibody used for detecting H3K9me2 was ab1220 (Abcam). 30 chromocentres were analysed to measure the ratio of H3K9me2 to DAPI, and the measurement was performed with Image J.

### Statistics and reproducibility

Fluorescent signal in each genotype shown in the figures were confirmed with two independent experiments, and the measurement data were generated once. Multiple plants from each genotype were used for phenotyping and microscopy.

### Reporting summary

Further information on research design is available in the Nature Portfolio Reporting Summary linked to this article.

## Supplementary information

Reporting Summary

Supplementary Tables Supplementary Tables 1 and 2.

## Source data

Source Data Extended Data Fig. 5 Unprocessed northern blots.

## Data Availability

Sequence data that support the findings of this study have been deposited in Gene Expression Omnibus with the accession codes GSE132005. The TAIR10 genome assembly was downloaded from TAIR (https://www.arabidopsis.org/) and the ColCEN assembly^32^ from https://github.com/schatzlab/Col-CEN. Previously published small RNA datatsets^18^ were used in this study (GSE52952). Source data are provided with this paper.
