# Supplementary material for: Retrotransposon addiction promotes centromere function via epigenetically activated small RNAs
Source: Nat Plants. 2024 Sep 2;10(9):1304–16. doi: 10.1038/s41477-024-01773-1 (PMC11410651; doi:10.1038/s41477-024-01773-1)
Supplement: Supplementary file 1 — Reporting Summary [file 41477_2024_1773_MOESM1_ESM.pdf]

nature portfolio

Corresponding author(s): Robert A Martienssen

Last updated by author(s): Jun 28, 2024

Reporting Summary

Nature Portfolio wishes to improve the reproducibility of the work that we publish. This form provides structure for consistency and transparency in reporting. For further information on Nature Portfolio policies, see our Editorial Policies and the Editorial Policy Checklist.

Statistics

For all statistical analyses, confirm that the following items are present in the figure legend, table legend, main text, or Methods section.

n/a	Confirmed	
☐	☒	The exact sample size (*n*) for each experimental group/condition, given as a discrete number and unit of measurement
☐	☒	A statement on whether measurements were taken from distinct samples or whether the same sample was measured repeatedly
☒	☐	The statistical test(s) used AND whether they are one- or two-sided<br>*Only common tests should be described solely by name; describe more complex techniques in the Methods section.*
☐	☒	A description of all covariates tested
☒	☐	A description of any assumptions or corrections, such as tests of normality and adjustment for multiple comparisons
☐	☒	A full description of the statistical parameters including central tendency (e.g. means) or other basic estimates (e.g. regression coefficient) AND variation (e.g. standard deviation) or associated estimates of uncertainty (e.g. confidence intervals)
☒	☐	For null hypothesis testing, the test statistic (e.g. *F*, *t*, *r*) with confidence intervals, effect sizes, degrees of freedom and *P* value noted<br>*Give P values as exact values whenever suitable.*
☒	☐	For Bayesian analysis, information on the choice of priors and Markov chain Monte Carlo settings
☒	☐	For hierarchical and complex designs, identification of the appropriate level for tests and full reporting of outcomes
☒	☐	Estimates of effect sizes (e.g. Cohen's *d*, Pearson's *r*), indicating how they were calculated

Our web collection on statistics for biologists contains articles on many of the points above.

Software and code

Policy information about availability of computer code

Data collection	The number of chromocenters per nuclei were analyzed from DAPI signals by Image J.
Data analysis	All analysis are described in the Methods section of the manuscript. The following softwares were used: Bismark v0.22.3; bowtie2 v2.4.1; Samtools v1.10; BEDtools v2.31.0; MACS2 v2.2.7.1; bonito v0.6.2; modkit v0.2.3; dorado v0.5.0; minimap2 v2.26-r1175; deeptools v3.5.0

For manuscripts utilizing custom algorithms or software that are central to the research but not yet described in published literature, software must be made available to editors and reviewers. We strongly encourage code deposition in a community repository (e.g. GitHub). See the Nature Portfolio guidelines for submitting code & software for further information.

Data

Policy information about availability of data

All manuscripts must include a data availability statement. This statement should provide the following information, where applicable:

- Accession codes, unique identifiers, or web links for publicly available datasets
- A description of any restrictions on data availability
- For clinical datasets or third party data, please ensure that the statement adheres to our policy

Sequence data generated for this study have been deposited in Gene Expression Omnibus with the accession codes GSE132005. The TAIR10 genome assembly was downloaded from TAIR (https://www.arabidopsis.org/) and the ColCEN assembly (Naish et al. 2021) from https://github.com/schatzlab/Col-CEN. Previously published small RNA datatsets (Creasey et al. 2014) were used in this study (GSE52952).

Research involving human participants, their data, or biological material

Policy information about studies with human participants or human data. See also policy information about sex, gender (identity/presentation), and sexual orientation and race, ethnicity and racism.

Reporting on sex and gender	NA
Reporting on race, ethnicity, or other socially relevant groupings	NA
Population characteristics	NA
Recruitment	NA
Ethics oversight	NA

Note that full information on the approval of the study protocol must also be provided in the manuscript.

Field-specific reporting

Please select the one below that is the best fit for your research. If you are not sure, read the appropriate sections before making your selection.

☒ Life sciences ☐ Behavioural & social sciences ☐ Ecological, evolutionary & environmental sciences

For a reference copy of the document with all sections, see nature.com/documents/nr-reporting-summary-flat.pdf

Life sciences study design

All studies must disclose on these points even when the disclosure is negative.

Sample size	No sample size calculation was performed for this study. Sample size is based on our experience and field convention for all experiments. For sequencing experiments, ChIP-seq was performed with two biological replicates following the guideline for ChIP-seq (https://genome.cshlp.org/content/genome/22/9/1813.full.html), and about ten pooled seedlings were used for one replicate, achieving reproducibility. For Whole Genome Bisulfite Sequencing, DNA sample was extracted from individual plant, and ten sterile and fertile plants for the epiRIL mapping and 4 suppressors for the suppressor screening were analyzed, succeeding to identify the causal region linked with the phenotype.
Data exclusions	No data was excluded from the analysis.
Replication	Two independent experiments were performed for ChIP-seq and obtaining microscope images, and all attempts at replication were successful.
Randomization	All samples were allocated randomly into experimental groups. Plants of different genetic backgrounds were grown on individual plates or pots, and were grown randomly in the growth chamber. For sample selection within the same genotype, plants were randomly selected.
Blinding	Phenotyping was performed without prior knowledge of the genotype. For other experiments, blinding was not applied as the experiments did not involve comparisons between treatment. For computational data analysis, blinding was not applicable as the same parameters were applied to all genotypes and data were processed in an unbiased manner.

Reporting for specific materials, systems and methods

We require information from authors about some types of materials, experimental systems and methods used in many studies. Here, indicate whether each material, system or method listed is relevant to your study. If you are not sure if a list item applies to your research, read the appropriate section before selecting a response.

Materials & experimental systems

n/a	Involved in the study
☐	☒ Antibodies
☒	☐ Eukaryotic cell lines
☒	☐ Palaeontology and archaeology
☒	☐ Animals and other organisms
☒	☐ Clinical data
☒	☐ Dual use research of concern
☐	☒ Plants

Methods

n/a	Involved in the study
☐	☒ ChIP-seq
☒	☐ Flow cytometry
☒	☐ MRI-based neuroimaging

Antibodies

Antibodies used	anti-H3K9me2 (ab1220, Abcam); anti-CENH3 (gift from Prof. S. Henikoff)
Validation	anti-H3K9me2 antibody is a ChIP-grade antibody tested in Arabidopsis and used in many previous publications (for example, PMIDs: 36127735, 34850679). anti-CENH3 (anti-HTR12) antibody was generated by Quality Controlled Biochemicals (Hoptkinton, MA) for the Talbert et al. 2002 study and was successfully used for ChIP-seq in Arabidopsis (PMID: 34762468, 38254210)

Dual use research of concern

Policy information about dual use research of concern

Hazards

Could the accidental, deliberate or reckless misuse of agents or technologies generated in the work, or the application of information presented in the manuscript, pose a threat to:

No	Yes
☒	☐ Public health
☒	☐ National security
☒	☐ Crops and/or livestock
☒	☐ Ecosystems
☒	☐ Any other significant area

Experiments of concern

Does the work involve any of these experiments of concern:

No	Yes
☒	☐ Demonstrate how to render a vaccine ineffective
☒	☐ Confer resistance to therapeutically useful antibiotics or antiviral agents
☒	☐ Enhance the virulence of a pathogen or render a nonpathogen virulent
☒	☐ Increase transmissibility of a pathogen
☒	☐ Alter the host range of a pathogen
☒	☐ Enable evasion of diagnostic/detection modalities
☒	☐ Enable the weaponization of a biological agent or toxin
☒	☐ Any other potentially harmful combination of experiments and agents

Plants

Seed stocks	ddm1-1; rdr1 (SALK_112300); rdr2 (SALK_059661); rdr6-11; kyp-4 (SALK_044606); tailswap cenh3;
Novel plant genotypes	epi-recombinant lines were generated by crossing rdr1 rdr2 ddm1 to rdr1 rdr2 RDR6/rdr6, as described in Methods.<br>Hairpin-complemented lines were performed by Agrobacterium mediated transformation, as described in Methods.<br>rdr1 rdr2 rdr6 ddm1 suppressors were generated with EMS screens, as described in Methods.
Authentication	The lines generated were genotyped by whole genome sequencing, their phenotypes were assessed by whole genome DNA methylation analysis, immuno-fluorescence imaging and cytogenetics. Further details are described in Methods.

ChIP-seq

Data deposition

☒ Confirm that both raw and final processed data have been deposited in a public database such as GEO.

☒ Confirm that you have deposited or provided access to graph files (e.g. BED files) for the called peaks.

Data access links<br>*May remain private before publication.*	https://www.ncbi.nlm.nih.gov/geo/query/acc.cgi?acc=GSE132005
Files in database submission	H3K9me2 IP and corresponding inputs for rdr1;2;6, rdr1;2;ddm1, rdr1;2;6;ddm1 and rdr1;2;6;ddm1;Cen5-Athila5<br>CENH3 IP and corresponding inputs for WT, rdr1;2;6, rdr1;2;ddm1, rdr1;2;6;ddm1 and rdr1;2;6;ddm1;Cen5-Athila5
Genome browser session<br>(e.g. UCSC)	NA

Methodology

Replicates	2 Biological replicates per genotype
Sequencing depth	All samples were sequenced paired-end 76bp, generating at least 20M raw reads each with minimum 80% mapping efficiency.
Antibodies	anti-H3K9me2 (ab1220, Abcam); anti-CENH3 (gift from Prof. S. Henikoff)
Peak calling parameters	MACS2 with default parameters (q value = 0.01)
Data quality	Data quality was assessed by mapping statistics, correlation between replicates and browser visualization confirming previously reported patterns.
Software	ChIP-seq analysis is described in Methods, using bowtie2 to map, and MACS2 to call peaks
