# Supplementary figures and images for: Retrotransposon addiction promotes centromere function via epigenetically activated small RNAs
Source: Nat Plants. 2024 Sep 2;10(9):1304–16. doi: 10.1038/s41477-024-01773-1 (PMC11410651; doi:10.1038/s41477-024-01773-1)

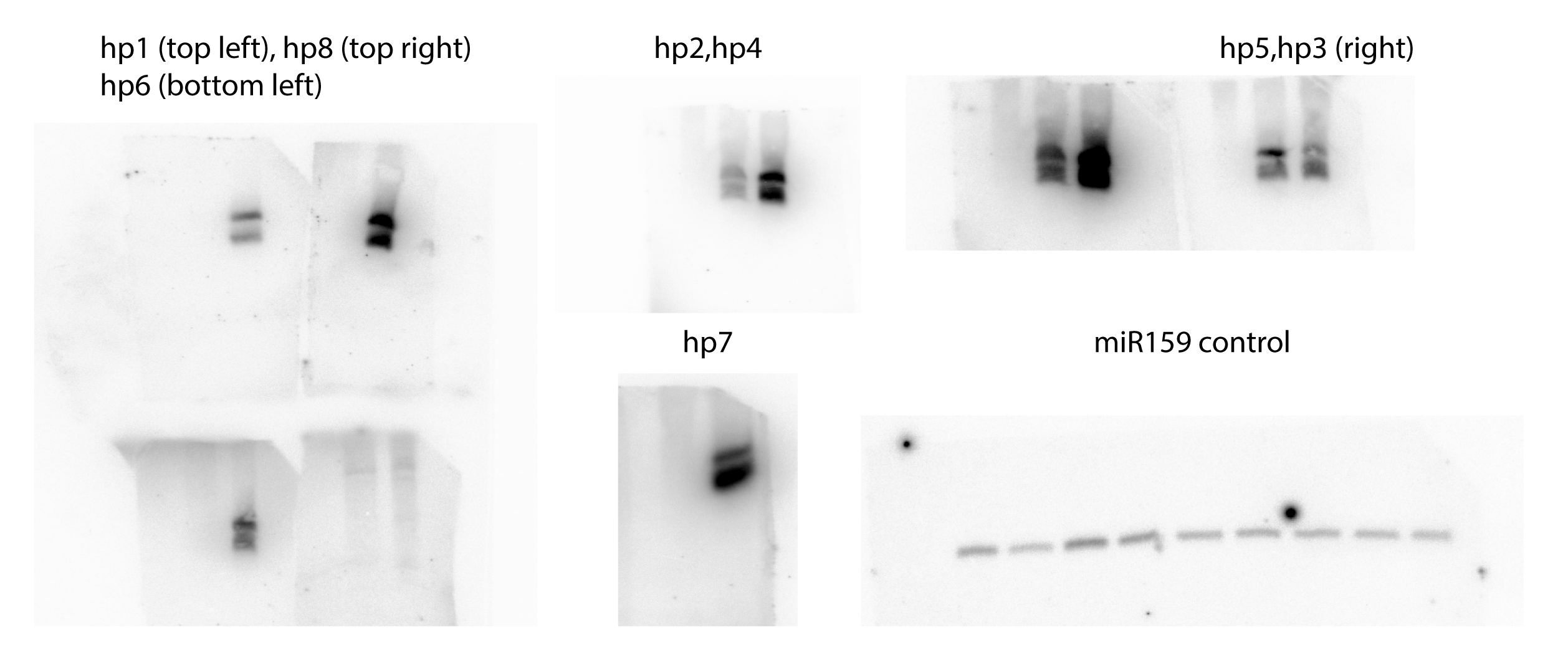

Supplement: Supplementary file 3 — Unprocessed northern blots. [file 41477_2024_1773_MOESM3_ESM.tif]
